# Participant engagement with a UK community-based preschool childhood obesity prevention programme: a focused ethnography study

**DOI:** 10.1186/s12889-019-7410-0

**Published:** 2019-08-08

**Authors:** Wendy Burton, Maureen Twiddy, Pinki Sahota, Julia Brown, Maria Bryant

**Affiliations:** 1 Clinical Trials Research Unit, Leeds Institute of Clinical Trials Research, University of Leeds, Leeds, LS2 9JT UK; 2 Institute of Clinical and Applied Health Research, Hull York Medical School, University of Hull, Cottingham Rd, Hull, HU6 7RX UK; 3 School of Clinical and Applied Sciences, Leeds Beckett University, City Campus, Leeds, LS1 3HE UK

**Keywords:** Children’s centres, Ethnography, Public health, Engagement, Obesity, Prevention

## Abstract

**Background:**

Children’s centres in the UK provide a setting for public health programmes; offering support to families living in the most disadvantaged areas where obesity prevalence is at its highest. Health, Exercise and Nutrition in the Really Young (HENRY) is an eight-week obesity prevention programme currently delivered in children’s centres across the UK. However, low participant engagement in some local authorities threatens its potential reach and impact. This study aimed to explore the factors influencing participant engagement with HENRY to describe where local intervention may support engagement efforts.

**Method:**

A focused ethnography study was undertaken in five children’s centres delivering HENRY across the UK. One hundred and ninety hours of field observations, 22 interviews with staff (commissioners, HENRY co-ordinators, managers and facilitators) and six focus groups (36 parents), took place over five consecutive days in each centre. The Consolidated Framework for Implementation Research (CFIR) was used to guide the observations and analysis of the data.

**Results:**

Three overarching themes described the factors influencing participant engagement with HENRY: local authority decision making around children’s centre programmes; children’s centre implementation of HENRY; and the participant experience of HENRY. The results indicate that factors influencing participant engagement with public health programmes begin at the commissioning body level, influencing children’s centre implementation and subsequently the experience of participants. Local authority funding priorities and constraints influence availability of places and who these places are offered to, with funding often targeted towards those deemed most at need. This was perceived to have a detrimental effect on participant experience of the programme.

**Conclusion:**

In summary, participant engagement is affected by multiple factors, working at different levels of the children’s centre and local authority hierarchy, most of which are at play even before participants decide whether or not they choose to enrol and maintain attendance. For programmes to achieve their optimal reach and impact, factors at the commissioning and local implementation level need to be addressed prior to addressing participant facing issues.

**Electronic supplementary material:**

The online version of this article (10.1186/s12889-019-7410-0) contains supplementary material, which is available to authorized users.

## Introduction

Children’s centres have the potential to support public health initiatives by providing a key setting for the delivery of public health programmes that are directed towards children and young families. They were launched in 2004 by the Labour government to build upon the success of the Sure Start initiative [1] which aimed to improve health and reduce inequalities by providing universal and targeted services to communities living in areas of high deprivation [2]. Children’s centres are required to provide a core offer of childcare, early year’s education, social support, and access to evidence based early years’ interventions. Public health programmes delivered in children’s centres are now accepted as a key strategy for obesity prevention [3–6].

In the UK, around one fifth of children start school overweight or obese, with the greatest prevalence in those from low socio-economic and ethnic minority backgrounds [7]. Ages 0–5 have been identified as a key life stage to target in obesity prevention efforts [8]. The development of health behaviours which influence obesity begin during this time and are influenced by parental habits and behaviours, media exposure and the home environment [9]. Therefore, obesity prevention programmes designed to change behaviours across the whole family offer great potential [10]. The children’s centre environment exposes families to health promoting policy and practice in addition to offering access to obesity prevention programmes; the combination of which may be related to better anthropometric outcomes for obesity prevention in preschool children [11]. However, there is currently limited evidence to support this outside of the US [12, 13]. Public health programmes delivered in children’s centres are mostly run by local practitioners that parents trust, maximising their potential for participant engagement [14]; but we know that obtaining participant engagement with public health programmes is a challenge [15, 16]. Many barriers to participant engagement with public health programmes are now understood; which include structural, social, cultural, and psychological level factors [17]. Additional barriers also exist when programmes comprise a parenting component, which has been associated with feelings of stigmatisation, fear and guilt [18–21].

Health, Exercise and Nutrition in the Really Young (HENRY) is an eight-week group parenting programme that is currently delivered in children’s centres located in around 10% of local authorities across the UK**.** The programme is aimed at young families with children aged 0–5 and takes a broad approach that includes some healthy eating and physical activity components, with equal focus on parenting skills and emotional well-being of the whole family [22]. Over 10,000 parents have participated since 2009. HENRY is underpinned by the Family Partnership Model [23] which uses the skills and qualities of practitioners to help parents and families overcome difficulties, build strengths and fulfil goals more effectively using a responsive and solution focused approach. The programme is predominately delivered by children’s centre staff (typically family outreach workers) who have attended a two day ‘core’ training session, followed by two days of HENRY facilitator training. The model of delivery is complex and varies by location. For example, the programme may be commissioned by local authorities, healthcare providers or third sector organisations who take ownership of implementing the programme e.g. deciding whether whole teams will be trained in the HENRY approach or just selected individuals. Typically, a HENRY coordinator will be appointed in each area who is responsible for coordinating local activity and providing support to facilitators. Earlier research indicates that the programme is well received by staff and parents [24, 25] and initial evidence provides some indication of a potential benefit, including positive changes in self-reported family diet, eating behaviours (e.g. frequency of family mealtimes) and parenting confidence [26, 27]. Evidence of effectiveness from randomised controlled trials is not yet available, though a feasibility study is currently underway [28]. However, uptake and retention vary across children’s centres and local authority areas which threatens its potential impact and reach. Akin to other community based programmes, process evaluation indicates that implementation targets are often not met. Data routinely collected by HENRY central office shows that centres delivering HENRY rarely recruit the target of a minimum of eight parents per course and only ~ 60% of parents attend at least five out of eight sessions. Poor enrolment and attendance are a widely reported challenge in group based childhood obesity interventions, with some paediatric weight management programmes reporting associations between poor engagement and obesity outcomes. For example, a recent study in the UK [29] examining participant attendance at a family based paediatric weight management group programme reported that completers (achieving > 70% attendance) achieved a significantly greater BMI loss than any of the other subgroups (initiators, late dropouts, low or high sporadic attendees). These findings were consistent with similar paediatric obesity treatment interventions in the U.S. [30, 31] Love et al. (2018) also reported that higher group numbers in a childhood obesity prevention programme in Australia promoted stronger group dynamics that encouraged greater outcomes. Further, a UK government report highlighted that low retention rates substantially increase programme delivery costs per participant which consequently impacts upon programme viability [32]. Thus, there is a need to create tailored methods to support local authorities, programme facilitators and parents to optimise parent engagement, therefore having the greater chance of population-based impact. Implementation research can help to unpick the factors influencing participant engagement with programmes [33]. Considering the behaviours of individuals involved in a programme’s implementation can also shed light on where participant engagement efforts could be improved [34]. This study aimed to explore the factors influencing participant engagement with HENRY to describe where local intervention may support areas to promote participant engagement with HENRY programmes or similar.

## Methods

We conducted a focused ethnography study which included field observations, focus groups and stakeholder interviews within the children’s centre setting. Ethnography allows researchers to obtain an in depth understanding of a phenomenon by immersing themselves in a setting and attaching meaning and interpretation to findings [35]. Focused ethnography is an adapted ethnography which is often used to allow for shorter periods of data collection, as existing knowledge and literature are drawn upon to narrow the focus of enquiry [36].

### Children’s Centre sampling

We applied a ‘positive deviant’ sampling frame [37, 38] to enable us to capture the potential local behaviours and beneficial practices from the centres and local authorities that support high level parent engagement, so that they could be applied elsewhere. This model works on the premise that some areas/groups have better outcomes than others. Improvements can therefore be efficiently made if uncommon, locally available and sustainable methods are universally applied. All centres that provided routine data from at least one completed HENRY programme during 2013–2014 were included in the analysis to identify centres deemed to be ‘positive deviants’ or ‘low engagers’ (see below).

There are inconsistencies and a lack of standardised definitions and criteria for engagement-related terminology [29]. The criteria used to categorise centres for our sampling framework was therefore based on, in part, recruitment and retention expectations set out by the HENRY central office, in addition to children’s centre deprivation rates, as historical evidence shows that it may harder to engage parents residing in areas of high deprivation [39]. The HENRY central office advocates that the ideal number of parents attending programmes is ten; with a minimum of eight, to ensure that there are a sufficient number of participants to facilitate strong group dynamics and to ensure delivery costs demonstrate value for money. To estimate children’s centre deprivation, Index of Multiple Deprivation (IMD) scores (http://tools.npeu.ox.ac.uk/imd/) were assigned to each centre. Positive deviants were defined as children’s centres that recruited at least eight participants to their most recent HENRY programme, retained at least 75% of parents for a minimum of five out of eight HENRY sessions, achieved participant compliance to intervention targets (measured via self-reported increase in fruit and vegetable intake as a proxy defined as ≥0.5 portion increase in fruit and vegetable intake), and located in areas of high deprivation as defined by IMD score (falling within the first or second quintile of area deprivation in England). Low engagers were defined as children’s centres that recruited < 8 participants on their most recent HENRY programme, retained < 75% of participantss for a minimum of five out of eight HENRY sessions, did not achieve participant compliance to intervention targets (measured via self-reported increase in fruit and vegetable intake as a proxy defined as < 0.5 portion increase in fruit and vegetable intake), and located in area of low deprivation as defined by IMD score (falling within the fourth or fifth quintile of area deprivation in England).

Routine HENRY programme data on recruitment and retention rates for the period 2013–2014 were available from 144 children’s centres. From these, 13 were identified as positive deviants and four as low engagers. All centres identified as positive deviants or low engagers were approached to take part in the study, but only those delivering a HENRY programme during the research (July 2015 – November 2015) were eligible to take part to allow for observations of HENRY practice; resulting in two positive deviants and two low engagers being included in the final sample. However, as the timeframe of the study allowed for a maximum of five children’s centres to be observed, a further centre was invited to take part, which met three out of the four positive deviant criteria (Recruited > 8 participants, ≥ 0.5 increase in fruit and vegetable intake and IMD scored within 4th quintile of deprivation) and delivered a HENRY programme during the study. This centre was deemed to be a ‘moderate’ engager. Therefore, five children’s centres delivering the HENRY programme were included in the research.

### Recruitment and consent

HENRY coordinators (who coordinate local HENRY activity within each local authority area) were approached by the central HENRY team to inform them that their local authority area had been selected to take part in the study. HENRY coordinators then contacted relevant centre managers to invite them to take part. Those who wanted to learn more about the study were contacted directly by the researcher (WB). Those happy to take part were asked to sign a children’s centre manager agreement (countersigned by a children’s centre lead from the local authority), permitting the researcher to conduct fieldwork in their centres.

Prior to the research taking place, all members of staff were notified that a researcher would be visiting the centre. During observations, visitors to the centres were advised of the researcher presence through notices placed in visible locations in the children’s centre reception. If visitors or staff did not wish to be observed, they were advised to let a member of staff/the researcher know, so that they would not be included in observations.

HENRY stakeholders (local authority commissioners, HENRY coordinators, centre managers and staff) from each participating children’s centre were invited to take part in an interview. Children’s centre managers, local authority commissioners and HENRY coordinators were invited directly by the researcher during initial communications to set up the research. The manager of each centre was then asked to nominate one HENRY facilitator from their centre to be interviewed and one staff member not trained to deliver HENRY programmes. Managers were free to select who they wanted to participate. Written informed consent was received before each interview took place.

Previous HENRY programme participants were invited to take place in focus groups via the children’s centre. Recruitment posters were displayed in each children’s centre to invite previous participants of HENRY to take part. Children’s centre managers were also asked to approach previous participants, including those who dropped out of the programme, to maximise numbers and ensure diversity of experiences. Informed written consent was obtained from all participants before the focus groups began.

Ethical approval was obtained from the University of Leeds School of Medicine Research Ethics Committee prior to undertaking the research.

### Data collection

Fieldwork took place over five consecutive days in each centre (25 days total). All ethnographic observation was undertaken by one researcher (WB). A second researcher (MB) also undertook observations in the first centre. Both researchers attended an advanced training course in doing ethnography prior to commencement of the study. Informal training on undertaking research observations was also provided by a member of the research team (MT). At the start of each visit the researchers met with the children’s centre manager to reiterate the rational for the study and discuss expectations of the visit i.e. what would be observed. Confidentiality and safeguarding procedures were also agreed.

The Consolidated Framework for Implementation Research (CFIR) [40] was used to guide the data collection. The CFIR is a conceptual framework derived from implementation theories which is used to facilitate the systematic evaluation of factors influencing programme implementation. In this study the CFIR constructs acted as an observation template during the children’s centre observations (see Additional file 1) and guided the interview topic guide (see Additional file 2). Utilisation of the CFIR constructs prompted consideration of multiple implementation levels within the children’s centre context e.g. individual, organisational and programme level characteristics, and their impact on participant engagement with HENRY.

### Ethnographical observations

At the start of each visit, the children’s centre timetable was reviewed to enable an observation schedule to be drawn up which structured the visit. This included observations of group sessions such as ‘stay and play’ and ‘rhyme time’ alongside more formal sessions e.g. parenting programmes (including HENRY). When group sessions were not being held at the centre, the researcher spent time sitting in the communal areas e.g. the reception area observing normal activity of the centre. A participatory ethnographical approach was used, whereby the researcher participated in the daily routines of the centres [41], to allow staff and family members to feel comfortable in their presence. For example, helping to set up for sessions, assisting in the crèche and taking lunch breaks with staff. The researcher referred to the CFIR observation template periodically throughout the day to map the observed behaviours, processes and culture against the CFIR constructs. During the first children’s centre visit, two researchers were present, and they met at the end of each day to discuss the findings and come to consensus about the CFIR constructs that observations were mapped to. This acted as quality assurance for the consistent use of the framework.

Ethnographical field notes were also collected. Brief field notes were taken discretely throughout the day and more complete notes were written once the researcher had left the centre. In these notes the researcher sought to capture the level to which families engaged with the children’s centre and other programmes delivered at the centre. Within the notes, the following details were captured: date; time and place of each observation; people present; details of what was observed; summaries of conversations; specific facts obtained; personal perceptions and interpretations of relevance to participant engagement; questions for future investigation; and initial thoughts of emerging key themes.

### Interviews and focus groups

Semi-structured interviews took place with local authority commissioners, HENRY coordinators, HENRY facilitators and centre managers and staff. All interviews were held at the participants’ place of work (i.e. children’s centre or local authority offices) and lasted between 60 and 90 min. All interviews were undertaken by WB. Focus groups were held within each children’s centre. Two were facilitated by WB and MB and three were facilitated by WB alone. All focus groups lasted between 30 and 60 min. The discussions centred around the participants’ experiences of HENRY, and their motivations to maintain or cease attendance (see Additional file 3 for topic guide). Field notes were made at the end of interviews and focus groups. Interviews and focus groups were audio-recorded, transcribed verbatim, and anonymised.

### Data analysis

Observations and the data analysis occurred concurrently. Data were imported in to NVivo data analysis software [42] to assist with coding and management. The aim of the analysis was to identify factors influencing participant engagement with HENRY across multiple levels of the children’s centre context. Deductive framework analysis [43] was applied to analyse the data, with the Consolidated Framework for Implementation Research used as the coding frame. In addition, commonly reported themes in the literature surrounding participant engagement with public health programmes from the participant perspective were added to the framework [21]. Where data fell outside of the a priori coding frame, inductive coding was applied. All data were coded by WB and a subset (10%) second coded by MT, with discrepancies in coding resolved by discussion. A third researcher was available to resolve disagreements, but was not needed. In accordance with principles of Framework analysis, the themes were presented as matrices to allow similarities and disparities between and within participant groups to be identified and aid interpretation. Ongoing analysis was discussed with the wider research team. Key concepts were then identified and overarching themes agreed.

## Result

Between July and November 2015, 190 hours of ethnographic observation were undertaken across five children’s centres (Table 1). Six focus groups were attended by 36 parents that had attended a HENRY programmee. No participants were recruited via the recruitment poster and therefore all parents were approached by the children’s centre manager to take part. The selection process used by managers on which parents to approach was not disclosed to the research team. All focus group participants had completed the programme (attended a minimum of five out of eight sessions). Twenty-two semi structured interviews were conducted with stakeholders comprising local authority commissioners, HENRY coordinators, HENRY facilitators and children’s centre managers and staff (Table 2). As described, children’s centre managers, local authority commissioners and HENRY coordinators all agreed to take part in interviews when approached by the researcher during study set-up. Centre managers then decided on which members of staff would take part, but did not disclose their decision making processes.

**Table 1:** Children’s Centre Characteristics

Centre	Geographic location	Area deprivation levels:	Positive deviant/low engager (criteria described above):
1	North West	5th Quintile of deprivation	Positive deviant
2	South East	1st Quintile of deprivation	Low engager
3	North West	5th Quintile of deprivation	Positive deviant
4	Midlands	2nd Quintile of deprivation	Low engager
5	Yorkshire	4th Quintile of deprivation	Moderate engager

5th Quintile of deprivation = most deprived area of the country

1st Quintile of deprivation = least deprived area of the country

**Table 2:** Interviews and focus group participant characteristics

Interviews	*n*	*n* = recruited from positive deviant, moderate engaging or low engaging centres	Gender (M/F)
Local authority commissioners	4	3 positive deviant; 1 moderate engager	3 Female; 1 Male
HENRY coordinators	4	2 positive deviant; 2 low engager	3 Female; 1 Male
Centre managers	5	2 positive deviant; 2 low engagers; 1 moderate engager	5 Female
HENRY facilitators	4	1 positive deviant; 2 low engager; 1 moderate engager	4 Female
Centre staff	5	2 positive deviant; 2 low engagers; 1 moderate engager	5 Female
Focus groups	*n*		Gender (M/F)
Parents that have previously attended a HENRY programme	36	11 positive deviant; 20 low engagers; 5 moderate engager	36 Female

The results of the study confirmed that factors influencing participant engagement with HENRY are present across multiple operational levels, consistent with the Consolidated Framework for Implementation Research domains: intervention characteristics, outer setting, inner setting, characteristics of individuals, and process. Thirteen of the thirty seven constructs were identified as influencing participant engagement (Table 3). In this paper, we describe the key CFIR constructs in relation to local authority commissioning, children’s centre implementation of HENRY and the participant perspective: implementation climate; available resources; evidence strength and quality; leadership engagement; external policies and incentives; personal attributes; and design quality and packaging.

**Table 3:** Consolidated Framework for Implementation Research constructs consistent with factors influencing participant engagement with HENRY

Intervention characteristics		Supporting quote
1	Adaptability: The degree to which an intervention can be adapted, tailored, refined, or reinvented to meet local needs.	Some HENRY facilitators described how they adapted programme material and activities to make sessions more engaging: “If I started talking about trans fats and saturated fats and hydrogenated fats, they would just switch off; “I don’t know what you’re talking about”. So what I do is, I bring a tin of beans in and I would just talk about good fats and bad fats.” (HENRY Facilitator)
2	Design quality and packaging: Perceived excellence in how the intervention is bundled, presented, and assembled.	The HENRY programme was perceived to be a high quality programme by commissioners, managers and centre staff. It was also highly acceptable to participants: “I think it’s excellent, excellent. My favourite thing is the fact that it’s so non-judgemental. It’s just, “this is the information, it’s up to you what you do with it”, and the fact, for somebody like me, who’s very stubborn, the fact that it’s not, “these are the rules and you have to do it”, it makes me much more likely to do it.” (Parent)
3	Cost: Costs of the intervention and costs associated with implementing the intervention including investment, supply, and opportunity costs.	The price of commissioning HENRY was described by some commissioners as being prohibitive: “The cost of HENRY is now getting prohibitive. I’ve really stayed true generally, I’ve moved my budgets around, I paid a lot for staff to go and train. But the actual cost of the licence and then the books that you have to buy, and then the resources after that, and actually, they’re pricing themselves out of the market” (Commissioner)
4	Evidence strength and quality: Stakeholders’ perceptions of the quality and validity of evidence supporting the belief that the intervention will have desired outcomes.	Commissioners described the value of participant outcome data to inform future commissioning decisions: “We’ve had one of our first reports back from HENRY which is invaluable to us here, you know, because then, when I’m going to commission and strategic meeting with heads of service around this work I can demonstrate back, this is what your staffing’s been doing, this is what a difference they’re making; and that helps it stay quite high on the agenda of people.” (Commissioner)
Outer setting		
5	External policies and incentives: External strategies to spread interventions, including policy and regulations (governmental or other central entity), external mandates, recommendations and guidelines,	Some centre managers described how external strategies influenced the programmes that were prioritised within centres: “Our targets are set by the local authority at an advisory board in the beginning of the year, so if you have a certain level of obese children in your area at reception class then you have to place HENRY or some sort of healthy living as a priority (Centre manager)
Inner setting		
6	Implementation climate: The absorptive capacity for change, shared receptivity of involved individuals to an intervention, and the extent to which use of that intervention will be rewarded, supported, and expected within their organization.	The local authorities differed in their implementation climate towards HENRY i.e. HENRY was more embedded in some areas than in others: “It feels like the integration of HENRY in [local authority] feels a little bit tepid” (Commissioner)
7	Leadership engagement: Commitment, involvement, and accountability of leaders and managers with the implementation.	Children’s centre managers directed the implementation of HENRY in their centres and therefore, obtaining their engagement with HENRY was important: “The manager is pretty crucial actually because my understanding is they’ve got a lot of freedom about what’s actually delivered in their centre. I think they actually need to be committed to HENRY” (Commissioner)
8	Available resources: The level of resources dedicated for implementation and on-going operations, including money, training, education, physical space, and time.	Funding constraints experienced at the local authority level impacted upon local implementation of HENRY, for example, the number of staff trained available to deliver the programmes: “We would like to offer the core training to all our children centres and health visiting staff but we just don’t have the funding” (Commissioner)
9	Access to knowledge and information: Ease of access to digestible information and knowledge about the intervention and how to incorporate it into work tasks.	Some members of staff expressed an interest in attending training on HENRY, or attending the HENRY programme itself to increase their knowledge around the programme: “I’d love to attend a course because I think attending a course gives you a feel of it and you can really promote it. If you’ve really enjoyed it you can promote it with such gusto.” (Staff member)
Characteristics of individuals		
10	Knowledge, & beliefs about the intervention: Individuals’ attitudes toward and value placed on the intervention as well as familiarity with facts, truths, and principles related to the intervention.	All interviewed stakeholders placed value on HENRY and felt that it was beneficial for families that attended: “I’ve seen HENRY have a really positive impact; really, really positive […] I think, if you have got a good facilitator, you have got a good group, the impact is massive, it really is.” (Centre manager)
11	Personal attributes: Personal traits such as tolerance of ambiguity, intellectual ability, motivation, values, competence, capacity, and learning style.	The personal attributes of staff members responsible for delivering HENRY were influential in motivating families to attend: “I think it’s once you know who’s going to be doing the course, that reels you in” (Parent)
Process		
12	Champions: Individuals who dedicate themselves to supporting, marketing, and ‘driving through’ an implementation, overcoming indifference or resistance that the intervention may provoke in an organization.	The HENRY facilitators ‘championed’ HENRY in their centres, dedicating themselves to promoting the programme: “I can’t do, say, be excited enough about HENRY. It really is a passion of mine since I’ve trained in it, and yeah, it should reach as many parents as possible. I think all parents should be offered the chance to go on it” (HENRY facilitator)
13	Engaging: Attracting and involving appropriate individuals in the implementation and use of the intervention through a combined strategy of social marketing, education, role modelling, training, and other similar activities.	Children’s centre staff in some centres approached people to attend based on their perception of how they could benefit from attending HENRY: “I say “oh we’ve got good HENRY course” if somebody’s talking about their baby […] they say “oh she’s such a fussy eater, she doesn’t eat very well, and I’m having such terrible trouble” and then I’ll say “we’ve got a HENRY course coming up, have you ever thought about that?” and then they’re like “what’s HENRY?” and then you explain it.” (Children’s centre staff member)

### Local authority commissioning

The factors influencing participant engagement with HENRY began at the commissioning body level (predominately local authorities) which appeared to have a spill-over effect to local implementation.

As local authority commissioning of HENRY did not take place within the children’s centre setting, it was not possible to observe this environment. Therefore, interviews with local authority commissioners were the primary source of data at this operational level but findings were supported with ethnographical observations.

### Implementation climate (how well HENRY is prioritised and supported in centres)

In interviews with commissioners it was clear that the extent to which HENRY was prioritised and supported in centres was driven by the level of local authority ‘buy-in’ and competing demands for funding. In both positive deviant centres, commissioners described how HENRY implementation was not top of the children’s centre agenda as services were becoming more directed to social care.

*“It feels like the integration of HENRY in [local authority] feels a little bit tepid. [ … ] It’s becoming increasingly difficult to engage in true prevention. I would argue that, the evidence that I see, is that when they talk about prevention they actually mean prevention of something else and they mean preventing children from going into social care which is a completely different model to a public health model.” (Commissioner; positive deviant centre)*

In contrast, the commissioner of the moderate engaging centre described HENRY as the cornerstone of their childhood obesity strategy:

*“Childhood obesity is a major issue at the moment so part of my responsibility was designing the city strategy around how we might help, prevent and manage childhood obesity, and HENRY is really a cornerstone of that strategy [ … ]. We have now got HENRY firmly embedded in [local authority] commissioning as a requirement for centres to run three groups per annum in each cluster. (Commissioner; moderate engager)”*

The commissioners from the low engaging centres also described how HENRY was well supported in their area:

*“We really like the approach, you know, it’s very collaborative. That’s our whole ethos really and that’s why we love it so much. I’ve put a lot of funding and commitment to making HENRY happen in [local authority] [ … ] It’s at the forefront of our health programme, absolutely.” (Commissioner; low engaging centre)*

In centres where commissioners indicated that HENRY was well supported within their area (moderate engaging and low engaging centres), ethnographic observations indicated that HENRY was well embedded into its principles and practice:

*“During observation of a nursery session, the room leader approaches me to talk about HENRY. She says that they have seen so many changes in the parents and the children as a result of them attending the programme. She also explains that within the centre itself, they adopt HENRY principles to help improve eating behaviours across the board, for example encouraging children to try new foods during meal times and encouraging parents to bring in different kinds of fruit for snack time. Every time a parent does bring in a piece of fruit for their child, they receive a counter to place in a jar as part of a ‘collective reward’ initiative which is a key element of the HENRY approach*.” (*Ethnographer observation; low engaging centre*)

In contrast, where commissioners described that HENRY was less supported, observations revealed a lack of HENRY promotion, and informal conversations with the staff indicated that they had little knowledge of the programme and were not involved in its implementation:

*“There are lots of display boards in the reception but there are no HENRY displays or HENRY posters. HENRY is also not included on the ‘What’s on’ guide [ … ] I am introduced to a parent engagement worker at a busy baby weigh in clinic. Her role is to meet and greet the parents, hand out leaflets showing what courses are going on, registers new people to the centre and occasionally offer one to one support and does home visits. I ask her if she tells parents about HENRY but she says doesn’t really have much to do with the programme, and that external teams, such as health visitors are the only ones that approach people to attend.” (Ethnographer observation; positive deviant centre)*

The level of support given to HENRY within the local authorities, as described by the commissioners, did not correspond to whether the attached children’s centres were classified as positive deviants, moderate engagers or low engagers; indeed, centres characterised as positive deviants from the quantitative data were significantly less engaged than those identified as low engagers.

### Available resources

Commissioners from the two positive deviant and the two low engaging centres described in interviews that the future of HENRY implementation in their areas was uncertain until funding streams had been secured. For example, one commissioner from a low engaging centre explained that funding which was previously available for HENRY from the public health budget had now being ring fenced for primary care initiatives:

*“In [local authority] the actual older person’s population is quite high and obviously there’s a lot of money goes towards looking after older people, as it should do. But you don’t necessarily get any additional funding for that, and the other thing is that there’s a lot of worried-well people possibly who are educated, middle-class and much more likely to go and ask for support about their health issues than people from deprived areas. So, actually, they take more time in terms of health services, so in terms of public health and the PCT, a lot of that money was taken away to support the acute hospitals”. (Commissioner; low engaging centre)*

In contrast, the commissioner from the moderate engaging centre described how they had been able to secure future funding for HENRY by aligning outcomes to the national healthy weight strategy:

*“I see it’s my responsibility to ensure that there’s enough funding for HENRY and that it’s in the right strategic plans and that the right people realise it’s a high priority and that it’s an effective way of working [ … ] What you do is you use your funding streams to deal with the wider determinants of health really [ … ] obviously we’ve got a national strategy, there’s a new heathy weight strategy coming out at the end of the year, so we should hopefully get colleagues set up and re-thinking and starting again about you know, well actually this is still a high priority, let’s see what we need to do to link with the launch of the national strategy.” (Commissioner; moderate engaging centre)*

Stretched HENRY budgets were described by commissioners as impacting upon local implementation and subsequently participant engagement. For example, commissioners attached to the two positive deviant children’s centres and a low engaging centre explained that they would not be able to provide any HENRY training for staff for the foreseeable future:

*“We would like to offer the core training to all our children centre and health visiting staff but we just don’t have the funding. I mean I would definitely recommend the core training to all staff, and in fact one of our community 0-19 teams which is the biggest said, ‘Oh, can our staff be trained?’ ‘Well if you can find some money’!” (Commissioner; low engaging centre)*

In interviews, staff members from the low engaging and positive deviant centres who had not been trained in the approach described how their lack of knowledge hindered recruitment efforts:

*“Even though I know a little bit about HENRY, you know, health and well-being etc. it’s like making a referral blindly. Because even though I’ve been online, on HENRY’s website, to read about it to make myself aware … I still feel like I’m a stranger! I have no idea what the HENRY is about” (Staff member, low engaging centre).*

However, no causal relationships can be drawn between a lack of resources and training provision as similar issues were described by commissioners attached to both positive deviant and low engaging centres.

### Evidence strength and quality

During interviews with commissioners, opinion was mixed regarding the existence of evidence demonstrating the positive effects of HENRY. The HENRY central office produce a report at regular intervals that is disseminated to local authorities containing participant data which includes self-reported changes to dietary and lifestyle behaviours. All commissioners in the sample described the importance of programme outcome data in demonstrating the impact of HENRY, which was key for strategic decision making. Commissioners from one positive deviant centre and both low engaging centres were happy with these reports and the outcomes they reported. However, one commissioner attached to a positive deviant centre could not recall seeing the report, and the commissioner attached to the moderate engaging centre remarked that the report was not as readily available as they would have liked:

*“There have been some glitches at HENRY so that flow of data has not been as quick to come as I’d have hoped [ … ] I think we need to get better data because it does help people to understand the impact of things and encourage further involvement.” (Commissioner; moderate engaging centre)*

Although this highlights the value of outcome data when considering commissioning decisions, such reports did not influence participant engagement or recruitment directly.

### Children’s Centre implementation of HENRY

### Leadership engagement

During interviews with commissioners, staff members and HENRY facilitators, the role of the centre manager was described as key in driving forward HENRY implementation in their centres:

*“The manager is pretty crucial actually because my understanding is they’ve got a lot of freedom about what’s actually delivered in their centre. I think they actually need to be committed to HENRY because obviously they’re having to give their staff time to actually run the group, it’s quite a big chunk of time, they’re giving their centre, they may be giving some crèche facilities. In terms of the actual overall organisation, they have to be behind it.” (Commissioner; low engaging centre)*

In three of the centres (positive deviant, moderate engager and low engager), managers described how they took an active role in directing recruitment efforts.

*“You’ve just got to invest the time in the recruitment, be well organised and plan it ahead of time. Don’t just rely on a family outreach worker to get that message out, work on it as a team; how are we gonna market it?, have a marketing strategy, bring in other people into that meeting when you’re looking at that, just don’t go it alone, you need your partners around you, so you need all the schools to be on board, the nurseries to be on board. Everybody need to be pushing it so that HENRY is known.” (Centre manager, moderate engaging centre)*

This suggests that manager buy-in could be a factor in achieving participant engagement, but as managers from all centres described their lead role in HENRY implementation, other factors are also likely to play an important role.

#### External policies and incentives

Children’s centre managers from the two positive deviant centres and a manager of a low engaging centre described in interviews that they adopted a targeted approach to recruitment (i.e. only the most vulnerable families approached to attend) in order to meet an expectation of Ofsted (Office for Standards in Education) and the local authority, that children’s centre services (including HENRY) prioritise the most vulnerable families in their area:

*“I’m responsible with the team to look at who gets a place, who’s a priority. We’re quite tied with crèche, so ratios can be a bit of a problem. In terms of how we recruit, we prioritise parents. Most are referred through health visitors or social care. So when we receive a referral from a health visitor, we’ll actually talk to them and say “What is the need? What is it they need?” So, for example, if the parent is just socially isolated they probably wouldn’t be priority to a parent who obesity runs in the family; who is eating six hash browns for breakfast; that kind of thing. So we really talk to who the referrers are to check, just to make sure we get the right families on the course.” (Centre manager; positive deviant centre)*

In one of the centres that adopted a targeted approach (positive deviant centre) the HENRY facilitator described in an interview how she felt the drive to target recruitment to more vulnerable families hindered participant engagement levels, as the parents invited to attend were often leading ‘chaotic lives’ and were not seeking to make diet and lifestyle changes. As such, they were reluctant to join in with group discussions, and did not engage with the solution focused approach which is central to the HENRY approach:

*“It’s been particularly hard with the targeted groups to get the parent’s solution focus stuff going. Because previously we’ve seen parents share things, whereas this time round we actually had to say, “this is your task for the next 6 weeks” because it’s not coming from anywhere else” (HENRY Facilitator; positive deviant centre)*

This view was supported in an observation of a HENRY session which took place of a ‘targeted group’:

*“I sat in on one of the HENRY sessions which was attended by a group of five parents who are ‘targeted’. The facilitator told me that the group should have been attended by seven people but two of them have not turned up. Three of the parents are morbidly obese and the other two are parents that I have met earlier in the week who are receiving support regarding unhealthy eating patterns. When the parents arrived, there was an awkward atmosphere as the facilitators were busy preparing the session and the parents did not engage with each other. The parents were very quiet during the session, one mother led most of the group discussion as the others remain quiet. One mother didn’t speak at all during the two hours. The parents did also not engage with the group activity. (Ethnography observation; positive deviant centre)*

During an observation of a breastfeeding support session at a different centre where targeting ‘at need families’ to HENRY was implemented (positive deviant centre), parents described in an informal conversation how they perceived HENRY to be ‘not for families like them’ and mainly for deprived families with obese children that required additional parenting support. Therefore, they felt that they did not need to attend classes like HENRY:

*“Whilst chatting to the mothers, one tells me that she has just had a gastric band fitted. The father is also overweight. They joke that they should go to a course like HENRY to assist with living a healthier lifestyle. Later on in the conversation she says she wouldn’t really want to go to HENRY as it was for deprived parents who lived in direct proximity to the centre, which is predominately high-rised social housing. Another parent joins in the conversation and explains that childhood obesity rates are high in their area, and therefore the parents of obese children need to attend courses like HENRY to teach them the importance of giving them a healthy diet and doing physical activity.” (Ethnographer observation; positive deviant centre)*

Although the majority of managers adopted a targeted approach, informal discussions with families and observations suggest that this approach does not result in better recruitment and retention rates, but may stigmatise families and deter attendance.

### Participant perspective

#### Personal attributes

In three out of the five centres (positive deviant, moderate engager and low engaging centre), previous participants of HENRY explained in focus groups that knowing and liking the facilitator of HENRY beforehand made them want to sign up:

*“I think it’s all in who approaches you. Because it was [name of facilitator], everyone knows him and he’s like the go to person if you need anything. He’s like everyone’s dad figure isn’t he, he’s there if you need some help, advice, someone to talk to, he’s there for you. If you need directing in one way or another, he’s there and he’ll do it … if the wrong person had approached me about it, I wouldn’t have done it.” (Parent; moderate engaging centre)*

The facilitators from these centres echoed this when describing what they felt was important when engaging participants to enrol:

*“The reason I think I do well [at recruitment and retention] is because I know personally how to meet their needs, and know where the parents are coming from, being sympathetic and basically knowing a little bit about their background. And you have to think, okay, this mum is coming with this baggage, and you work alongside with that baggage and you support them.” (HENRY facilitator; low engaging centre)*

Previous participants of HENRY from all centres also described in focus groups how the personal characteristics of the facilitator had made the programme more engaging:

*“She discussed her problems with us also; like she used to have a wine at the end of the day and she wanted to lessen her wine consumption at night and she made dinner for herself and she really drank on the night, she told us that. So it was like good to look to her and see that ok she has problems also. So it was like you know, sort of a bit of guidance like ok, she has her problems and gets over that and so we can, you know, a role figure for us. If she’s going through that then we can also, and we can open up to our problems” (Parent; positive deviant centre)*

Parents from a moderate engager, positive deviant and a low engaging centre also described how the facilitator had made a wider impact in their lives:

*“She kept pushing us to you know, get out a bit, experience more stuff outside instead of just being indoors all the time. The housework does not stop after that. You know it carries on, on, on. It’s just good to know sometimes, just get out and you know, if gives you a fresh mind as well, so it really helped me a lot.” (Parent; low engaging centre)*

However, although the personal characteristics of the facilitator clearly influenced the engagement levels of some individuals, there are no clear links between the facilitator and centre level engagement levels.

#### Design quality and packaging (interpreted as programme acceptability)

Focus group participants from all centres described how much they had enjoyed attending HENRY and described the impact it had made in their lives, suggesting that the programme is highly acceptable:

*“Yeah it was very very good. My kid routine was very rubbish before HENRY, he liked to watch all time i-pad and TV, and after attending this I know how much time he can watch that and it was very very helpful. And his eating routine is now very good because of that and er lots of things good for me.” (Parent; low engaging centre)*

It also appeared that the solution focused approach and support received from peers was instrumental in maintaining engagement:

*“We all came up with our problems and then we tried to solve them; whether it worked or not it was really good. It was like a real mix of people, and people were like ‘oh no, he’s still only eating chips and the group were trying to come up with something again.” (Parent; positive deviant centre*)

However, as all focus group participants had completed the HENRY programme, it is unclear whether drop outs (in particular from the low engaging centres) found the programme unacceptable or if other factors were at play.

## Discussion

This study describes some of the main factors influencing engagement with a public health community-based intervention: the HENRY group programme. Prior work in this area has highlighted factors associated with barriers and facilitators to engagement at the participant level which are consistent with these findings, such as the role of the facilitator, group dynamics and programme delivery style [44–49]. A novel finding from our study is the importance of establishing and maintaining support for the intervention from all levels of the organisation.

A key finding at all centres, was that the starting point for initiating participant engagement predominantly began at the level of local authority which determined how much priority and investment was given to its implementation. This then impacted upon centre level practices and how embedded into the children’s centre the programme was, which ultimately shaped the participant experience and consequent engagement.

Data collection took place at a time of great uncertainty for HENRY centres. In 2011, the UK Government directed children’s centres to target services to those families most in need of their support but acknowledged that universal access was also important to avoid stigma and to promote participant engagement [50]. Since then however, budgets have significantly decreased, making it difficult for many centres to offer a ‘universal service’.

Obesity services are not mandated or statutory for local authorities so commissioning of services is dependent on funding levels and local priorities. Community level data are used to specify local need, so the level of investment is dependent on whether their objectives are consistent with the local authority’s identified public health priorities and the degree to which commissioners perceive them to be value for money. Evidenced based programmes are increasingly becoming a funding requirement in public health [51] and it is known that programmes found to be effective are more likely to be supported [52]. However, the evidence base or these interventions is still being developed.

The targeting of programmes such as HENRY to those deemed to be at greatest need may be out of necessity, but the implications of this are unknown. Parents visiting children’s centres prefer to attend when services are offered universally to allow a greater choice of services and to reduce feelings of stigma [53]. Further, broader provision allows more families to receive support enabling population level benefits [54]. A further implication of restricting provision is defining which families would most benefit, as this can be open to interpretation [50]. Moreover, determining the families most ‘in need’ may be particularly difficult when, as our study has shown, staff members responsible for recruitment may be unsure of what the programme offers.

Managers were a central gatekeeper for promoting participant engagement. This supports what has been reported previously in the literature; that centre managers are responsible for decision making around the programmes they deliver and offer their support to programmes that address local need [55]. Further, mangers are more likely to engage with programmes if they are found to be acceptable to both practitioners and the participants that attend.

The role of the programme facilitator is known to be a key factor in achieving participant engagement [44–46]. The facilitator is responsible for creating a non-judgemental and empathetic environment in addition to ensuring that programmes are facilitated as specified, and with sensitivity to manage different characteristics within the group [21]. The ongoing assessment of programme acceptability is also important, so that participants are more likely to maintain attendance and achieve greater outcomes. [56] This is particularly significant when the programme is delivered to a range of population groups.

### Strengths and limitations

The ethnographic approach, informed by a strong theoretical model provided an in depth understanding of the cultural, organisational, and personal participant engagement, and allowed key findings and interpretation of the data to be triangulated across different data sources and types. The focused ethnography allowed key questions to be selected *a priori* and the existing literature drawn upon, allowing for shorter periods of data collection and more centres to be visited.

We used routinely collected quantitative metrics to develop a sampling framework to recruit centres based on participant recruitment and retention data to understand why some centres achieve higher levels of participant engagement than others. This framework categorised centres as demonstrating high, moderate, or low levels of engagement. However, our interview and ethnography data show this approach was not useful in identifying those centres most or least engaged with the programme. This may be because we used current recruitment data which provided a snapshot of recruitment in centres, but did not account for historical recruitment patterns. Centres categorised as low recruiters may have previously been good recruiters, but are now affected by funding restraints and closures, giving a misleading picture. Future studies should consider using a more nuanced approach to identifying cases.

Focus group participants and staff interviewees were recruited via centre managers, which may bias the findings, and although independence of the researcher from the HENRY programme and the children’s centre was made explicit, some participants may have not wanted to speak unfavourably about the programme or centre [57]. Focus group participants in this study were previous HENRY programme attendees, so unsurprisingly found the programme and recruitment process acceptable. No-one with a negative experience of HENRY volunteered to participate in focus groups, so less is known about why families do not attend, or drop out, although participants did provide some insights.

As the research team consisted predominately of nutritionists with an interest in obesity and public health, the direction of the research was geared towards seeking answers on how programmes can be maximised to achieve population level change. Therefore, the ethnographic data gathered for this study was analysed and interpreted through the lens of identifying where implementation processes could be optimised to promote participant engagement. It is therefore acknowledged that sociological and cultural factors outside of the children’s centre context are likely to influence whether participants do or do not engage with public health programmes which have not been reported here that warrant further exploration.

## Conclusions and future implications

In summary, this study has established that multiple factors, working at different levels, exist across the children’s centre and local authority hierarchy that work together to influence participant engagement to an obesity prevention programme. These factors are at play even before participants decide whether or not they choose to enrol and maintain attendance; thus, in order for programmes to achieve their optimal reach and impact, factors at the commissioning and local implementation level need to be addressed prior to addressing participant facing issues. Specifically, our results would suggest that participant engagement strategies should be aimed at a minimum of three levels. The first level should include strategies aimed at obtaining the commissioning body ‘buy-in’ to ensure that programmes receive adequate financial support for their effective implementation. The second should focus on optimising manager support of programmes to ensure that local processes are implemented that promote recruitment and retention, including provision of universally available services. Finally, the third level could focus on the participant’s experience including how they are made aware of the programme, the level of information they receive before enrolling on to the sessions, and the importance of having an engaging and skilled facilitator. To support this, programme providers need to evidence measurable change in outcomes that are of value and importance to the commissioning local authority and centre managers, as well as parents. Further, continued assessment of acceptability and participant engagement would allow programmes to adapt accordingly enabling a range of participant groups to benefit.

## Additional files

Additional file 1: Observation template. (XLSX 16 kb)

Additional file 2: Interview topic guide. (DOC 34 kb)

Additional file 3: Focus group topic guide. (DOCX 20 kb)

## Data Availability

Not applicable.

## Abbreviations

CFIR: Consolidated Framework for Implementation Research
HENRY: Health Exercise Nutrition for the Really Young
IMD: Index of Multiple Deprivation
