# Supplementary material for: Participant engagement with a UK community-based preschool childhood obesity prevention programme: a focused ethnography study
Source: BMC Public Health. 2019 Aug 8;19:1074. doi: 10.1186/s12889-019-7410-0 (PMC6688247; doi:10.1186/s12889-019-7410-0)
Supplement: Supplementary file 2 — Interview topic guide. (DOC 34 kb) [file 12889_2019_7410_MOESM2_ESM.doc]

***Additional file 2 Interview topic guide***

1. Can you describe your role in the implementation of HENRY?
2. How engaged are you with HENRY?
3. Does HENRY meet your needs? Is there an alternative?
4. How do you feel about the style/delivery/content?
5. Does this fit with your centre ethos?
6. Can you describe what you perceive to be the evidence base around HENRY?
7. Are there any barriers to delivering HENRY?
8. What sort of impact does HENRY have?
9. After the area/centre began delivering HENRY, were any changes implemented?
10. Who has attended training on HENRY in your area/centre? Why were they selected?
11. In some areas, centres struggle to engage participants to HENRY, why do you think that might be?
12. How important is the HENRY coordinator/HENRY facilitator/centre staff/centre manager to engaging parents with HENRY?
13. Do you monitor recruitment and retention? Why?
14. Are there any goals or incentives for staff to recruit?
15. Do you compare your recruitment and retention rates with any other centres/areas?
