# Supplementary material for: Participant engagement with a UK community-based preschool childhood obesity prevention programme: a focused ethnography study
Source: BMC Public Health. 2019 Aug 8;19:1074. doi: 10.1186/s12889-019-7410-0 (PMC6688247; doi:10.1186/s12889-019-7410-0)
Supplement: Supplementary file 3 — Focus group topic guide. (DOCX 20 kb) [file 12889_2019_7410_MOESM3_ESM.docx]

***Additional file 3 Focus group topic guide***

1. How did you learn about the HENRY programme?
2. What made you want to enrol?
3. How many sessions did you attend? If you missed a session why was this?
4. What did you think HENRY was when it was first recommended/you first saw the poster?
5. Did anyone describe HENRY to you before you signed up?
6. In some areas, HENRY struggles to get enough parents to enrol on the course. Why do you think that is?
7. Why you think some families fail to attend after they have enrolled?
8. What did you think about the HENRY sessions/content/delivery?
9. Were there any messages/tasks that you found hard to do or maintain?
10. What qualities are important to you in a facilitator?
11. What did HENRY do for you?
12. Would you recommend HENRY to other people?
13. Could anything about HENRY be improved at all?
